# Efficacy of the Monte Carlo method and dose reduction strategies in paediatric panoramic radiography

**DOI:** 10.1038/s41598-019-46157-0

**Published:** 2019-07-04

**Authors:** Chena Lee, Bora Park, Sam-Sun Lee, Jo-Eun Kim, Sang-Sun Han, Kyung-Hoe Huh, Won-Jin Yi, Min-Suk Heo, Soon-Chul Choi

**Affiliations:** 1 Department of Oral and Maxillofacial Radiology, Yonsei University College of Dentistry, Seoul, Republic of Korea; 2 Department of Oral and Maxillofacial Radiology and Dental Research Institute, School of Dentistry, Seoul National University, Seoul, Republic of Korea

**Keywords:** Paediatric research, Paediatric research, Panoramic radiography, Radiography

## Abstract

Monte Carlo (MC) simulation is a simpler radiation dose assessment method than the conventional method, thermoluminescent dosimetry (TLD). MC simulation and TLD were compared as tools to evaluate the effective dose from paediatric panoramic radiography. Various exposure conditions and machine geometries were simulated using the MC method to investigate factors resulting in effective dose reduction. The effective dose of paediatric panoramic radiography was obtained using an MC simulation and its reliability was verified by a comparison with the value obtained using TLD. Next, 7 factors determining the effective dose in the MC simulation were input with 6 equally-spaced values, and a total of 36 simulations were performed to obtain effective dose values. The correlations between each dose-determining factor and the resulting effective dose were evaluated using linear regression analysis. The TLD-measured dose was 3.850 µSv, while the MC simulation yielded a dose of 3.474 µSv. Beam height was the factor that most strongly influenced the effective dose, while rotation angle and focus-to-patient distance were the least influential factors. MC simulation is comparable to TLD for obtaining effective dose values in paediatric panoramic radiography. Obtaining panoramic radiography with a short beam height can effectively reduce the dose in paediatric patients.

## Introduction

The human body is known to be more sensitive to radiation at younger ages, because the cells comprising the body organs have a high potential for differentiation and actively divide in children^1,2^. In paediatric patients, the impacts of radiation remain present for a longer period of time after exposure than is the case for adult patients. Therefore, making efforts for dose reduction considering the balance of risks versus benefits is especially critical for young patients. Previous research reported that diagnostic X-rays may increase the incidence of leukaemia and brain cancer and may have the potential to trigger the onset of any type of cancer in children^3^. Even if the expected amount of radiation is minor in dental panoramic radiography, efforts to apply it carefully and to reduce the dose are essential for paediatric patients^4,5^.

Assessments of the effective dose of panoramic radiography have generally been made using thermoluminescent dosimetry (TLD) with human phantoms, although TLD involves certain inconveniences and errors. Some recent studies have attempted to replace TLD with optically stimulated luminescent dosimetry^6^, the advantages of which include less delicate equipment and more time-effective preparation and reading processes.

Other researchers have tried to obtain effective dose estimates using computer calculations based on Monte Carlo (MC) simulations^7–9^. The MC algorithm is a method of calculating the effective dose by simulating the interaction of each X-ray photon with body tissue. Although this method has been established to be accurate in the medical field^10^, it is not yet widely used with dental diagnostic X-rays. However, a recent study showed its accuracy and applicability in dental panoramic radiography compared to TLD^9^. As far as the authors know, no study in the English-language literature has reported the accuracy of MC simulations of the effective dose in dental diagnostic X-rays in paediatric patients.

Therefore, this study was conducted to evaluate the effective dose calculated with the MC method compared to the measurements obtained using TLD in paediatric panoramic radiography. In addition, various exposure conditions and machine geometries were simulated with the MC method to investigate the factors contributing to effective dose reduction.

## Methods

### TLD measurements

**Figure 1:**
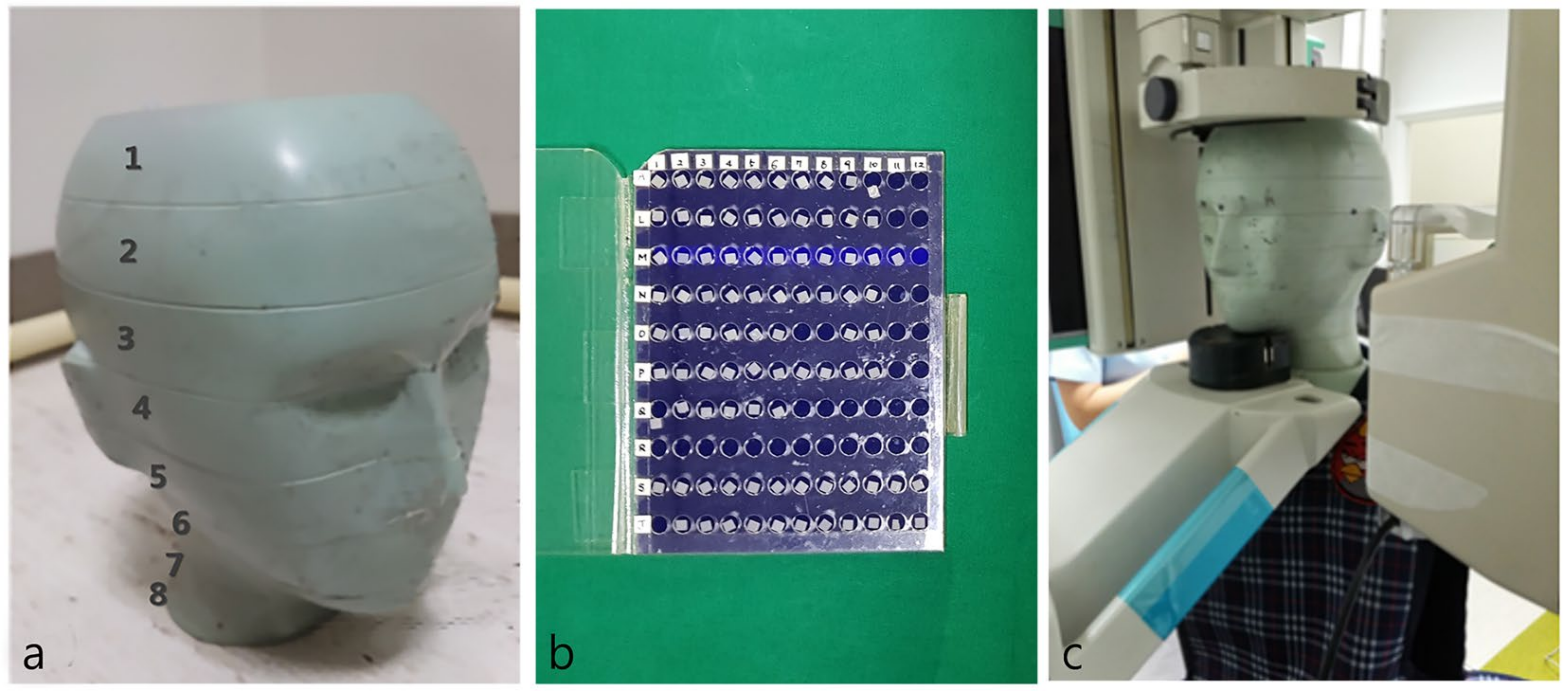
(**a**) Head and neck components of 5-year-old anthropomorphic phantom composed of 8 slices. (**b**) Thermoluminescent dosimetry (TLD) chips. (**c**) The phantom embedded with TLD chips was exposed to radiation using the paediatric mode of panoramic radiography.

The head and neck components of a 5-year-old anthropomorphic phantom (ATOM® dosimetry phantom, model 705-D, CIRS, Norfolk, VA, USA), consisting of 8 slices with holes for TLD housing, were used (Fig. 1). The phantom was constructed to have equivalent tissue density to a human body measuring 110 cm in height and 19 kg in weight.

TLD utilizes dosimeters that store radiation as energy and release it as light when stimulated by heat. The intensity of emitted light is converted into a value indicating the radiation dose via the reading unit. In this study, 48 pre-calibrated LiF TLD-700 chips (LiF 7: Mg, Ti) measuring 1/8 inches × 1/8 inches × 0.03 inches, with assigned identification (ID) codes, were used. Calibration was performed by a nationally certified company (ILJIN Radiation Engineering Co., Ltd, Gyunggi-Do, Korea) that maintains personal TLD badges through the following process. Dosimeters were exposed to 5612.7 µGy of radiation. Each dosimeter was read using a RADOS RE-1 reader (Rados Technology, Turku, Finland). The data were recorded and the sensitivity of each TLD detector was obtained. Since TLD was read as gamma energy which presents 1.25 times higher sensitivity compared to the x-ray, a correction factor of 0.8 was multiplied to normalize the value. TLD detectors with <±5% error were selectively used for this experiment.

Sixteen anatomic sites were selected and 3 TLD chips were placed at each site to minimize error. The dosimeter placement procedure was in accordance with previous studies^6,11^. The TLD chip ID and the anatomic site of each organ are summarized in Table 1.

**Table 1:** Specific location of the thermoluminescent dosimetry (TLD) chips and their identification (ID) codes according to each anatomic site of the RANDO phantom.

Organ		Anatomic site	Location number	TLD chip ID
			Slice (hole)	
Bone marrow and bone		Anterior calvarium	2 (1)	H3, H7, H8
		Left calvarium	2 (2)	G4, G11, G12
		Right calvarium	2 (3)	F6, F12, G3
		Right ramus	6 (19)	AS1, AS4, AS7
		Left ramus	6 (17)	P6, Q1, R1
		Center cervical spine	8 (21)	N2, O1, O3
Brain		Midbrain	3 (6)	D2, E2, E3
		Midbrain	3 (7)	E11, F2, F5
		Pituitary	4 (12)	K3, K6, K12
Oesophagus		Oesophagus	8 (21)	A6, L2, L3
Salivary gland		Right parotid gland	6 (19)	AS1, AS4, AS7
		Left parotid gland	6 (17)	P6, Q1, R1
		Right submandibular gland	6 (19)	AS1, AS4, AS7
		Left submandibular gland	6 (17)	P6, Q1, R1
		Center sublingual gland	6 (18)	O4, O7, O8
Skin		Right lens of eye	4 (15)	B3, B4, B9
		Left lens of eye	4 (16)	C6, C7, C11
		Left back of neck	6 (20)	AS10, J3, J8
Thyroid		Left thyroid	8 (22)	M7, N1, H10
		Right thyroid	8 (23)	M1, M6, H9
Remainder tissue^a^	Extrathoracic airways	Right maxillary sinus	4 (15)	B3, B4, B9
		Left nasopharynx	4 (12)	K3, K6, K12
		Right parotid	6 (19)	AS1, AS4, AS7
		Left parotid	6 (17)	P6, Q1, R1
		Right submandibular gland	6 (19)	AS1, AS4, AS7
		Left submandibular gland	6 (17)	P6, Q1, R1
		Center sublingual gland	6 (18)	O4, O7, O8
		Oesophagus	8 (21)	A6, L2, L3
	Lymph nodes and muscles	Right parotid gland	6 (19)	AS1, AS4, AS7
		Left parotid gland	6 (17)	P6, Q1, R1
		Right submandibular gland	6 (19)	AS1, AS4, AS7
		Left submandibular gland	6 (17)	P6, Q1, R1
		Center sublingual gland	6 (18)	O4, O7, O8
		Left thyroid	8 (22)	M7, N1, H10
		Right thyroid	8 (23)	M1, M6, H9
	Oral mucosa	Right parotid gland	6 (19)	AS1, AS4, AS7
		Left parotid gland	6 (17)	P6, Q1, R1
		Right submandibular gland	6 (19)	AS1, AS4, AS7
		Left submandibular gland	6 (17)	P6, Q1, R1
		Center sublingual gland	6 (18)	O4, O7, O8

^a^Among the 14 remainder tissues, the extrathoracic airways, lymphatic nodes, muscle, and oral mucosa were included for calculating the maxillofacial dose.

The TLD-embedded phantom was exposed to panoramic radiography using an Orthopantomograph OP100 (Instrumentarium Imaging, Helsinki, Finland). Paediatric mode was selected, with exposure conditions of 66 kVp, 8.0 mA, and 16.8 seconds. The exposure conditions were chosen to correspond to the standard conditions used for 5-year-old children at Seoul National University Dental Hospital. Exposures were performed 3 times and averaged values were used to minimize error (Fig. 1).

The TLD chips were left for 24 hours, and the energy level stored in the chips was then read with a RADOS RE-1 reader (Rados Technology, Turku, Finland). Three unexposed chips were read to determine the amount of background radiation, which was subtracted from the results of the exposed chips. The absorbed dose of each anatomic site was obtained by averaging the measured value of the 3 chips in micrograys (µGy). The values from each anatomic site were integrated into the organ dose considering the tissue-irradiated fraction of the head and neck (Table 2). For example, the bone marrow dose was obtained by considering its distribution in the calvarium (11.6%), mandible (1.1%), and cervical spine (2.7%)^12^. Additionally, the bone surface dose was obtained by multiplying the bone marrow value by the bone-to-muscle attenuation ratio, which was defined as −0.0618 × kV(p) × 2/3 + 6.9406^13^. The exposure of the skin, muscle, and lymph nodes was estimated to account for 5% of the total body tissue. The exposure fraction of the oesophagus was estimated as 10%. Other tissues of interest were counted as 100%. The individual organ doses were then integrated into the effective dose considering the tissue weighting factors suggested in 2007 by the International Commission of Radiological Protection (Table 2)^14^.

**Table 2:** Fraction of head and neck tissue irradiated during X-ray examinations of paediatric patients and tissue weighting factors.

Organ		Fraction irradiated (%)^12^	Tissue weighting factor^14^
Bone marrow		15.4	0.12
Calvarium		11.6	
Ramus		1.1	
Cervical spine		2.7	
Bone^a^		16.5	0.01
Calvarium		11.8	
Ramus		1.3	
Cervical spine		3.4	
Brain		100	0.01
Oesophagus		10	0.04
Salivary gland		100	0.01
Parotid gland		100	
Submandibular gland		100	
Sublingual gland		100	
Skin		5	0.01
Thyroid		100	0.04
Remainder tissues	Extrathoracic airways	100	0.12
	Lymph nodes	5	
	Muscles	5	
	Oral mucosa	100	

^a^Bone = bone marrow dose × bone/muscle mass energy absorption coefficient ratio (MEACR), MEACR = 0.0618 × 2/3 kVp + 6.9406^13^.

### MC simulation

Monte Carlo (MC) simulation is an algorithm for predicting the interactions of X-ray photons with a complex medium, such as the human body^15^. When the appropriate physical and mechanical information is given, the organ-absorbed dose and effective dose can be calculated using computer software based on this algorithm.

### Dose assessment in general conditions

For the MC simulations, PCXMC20Rotation (STUK, Helsinki, Finland), a supplemental program of PCXMC 2.0, was used. The virtual phantom of a 5-year-old in the program was 19 kg in weight and 109.1 cm in height.

**Figure 2:**
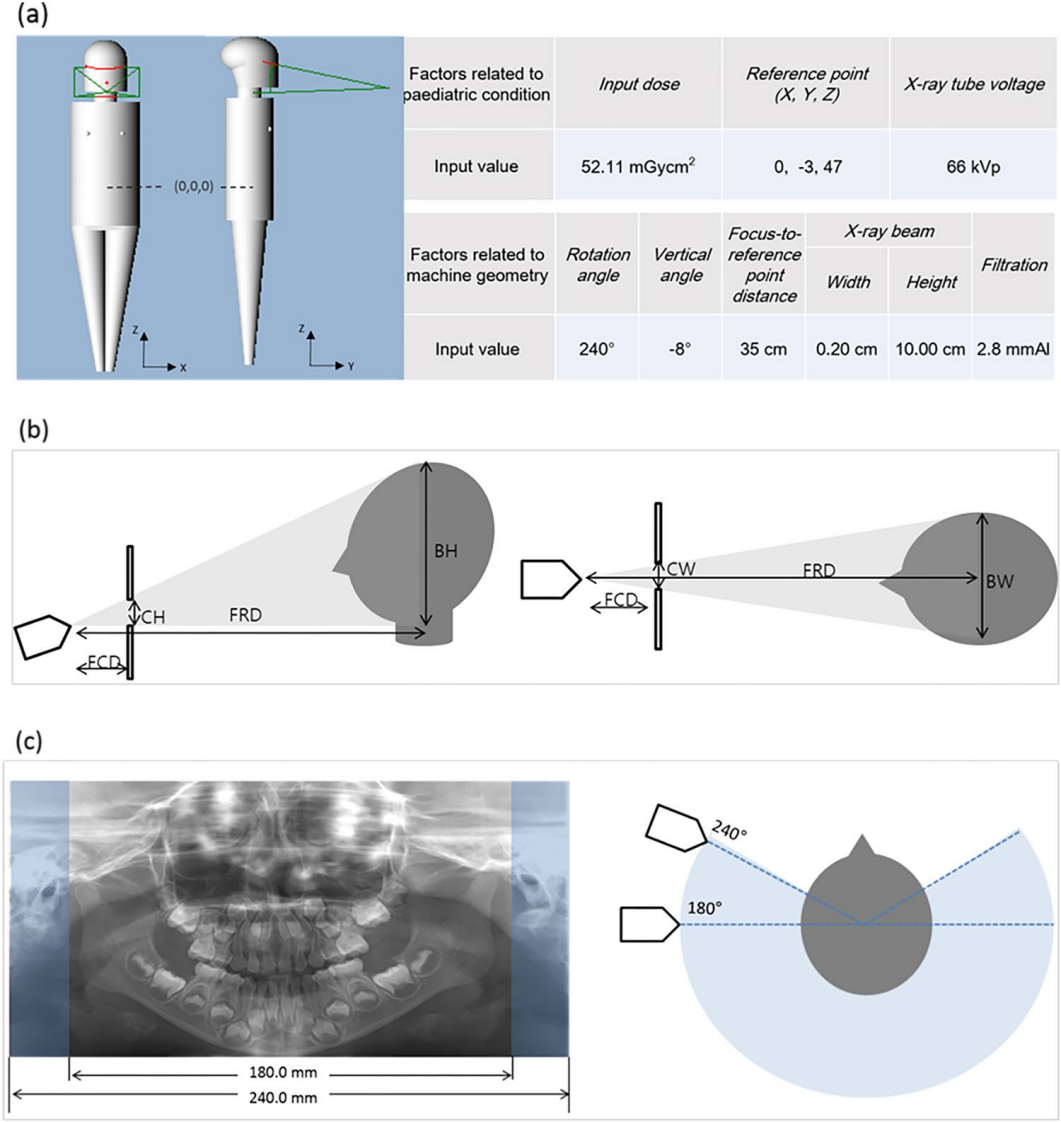
(**a**) The virtual 5-year-old phantom and the input values of the factors used to perform the Monte Carlo simulation. Schematic view of beam width, height (**b**), and rotation angle of the X-ray source (**c**) in panoramic radiography. Beam height and width were calculated based on the source-collimator distance, source-patient distance, collimator height, and collimator width. (FCD, focus-to-collimator distance; CH, collimator height; FRD, focus-to-reference distance; BH, beam height; CW, collimator width; BW, beam width). The rotation angle in panoramic radiography is correlated with the image length measured with a digital calliper. According to the manufacturer’s specifications, the rotation angle was 240°, which produced images of 240.0 mm in length. The possible minimum image length covering the lateral pole of the condyle was measured as 180.0 mm, which would correspond to a rotation angle of 180°.

To obtain the absorbed dose and effective dose, the program required proper input values to be entered for the following factors: exposure dose, reference point, X-ray tube voltage, rotation angle, vertical angle of central ray, focus-to-reference distance (FRD), X-ray beam width/height, and filtration. The input values selected for the MC simulation corresponded to the same conditions as the TLD measurement method. The input values for the factors related to paediatric patients were determined as described below (Fig. 2a).

#### Exposure dose

The dose-area product (DAP, mGy·cm^2^) is used for assessing the radiation dose of a diagnostic X-ray unit. The DAP of panoramic radiography in paediatric examination mode was measured using a DAP meter (Diamentor M4-KDK, PTW, Freiburg, Germany). The DAP meter was composed of an ionization chamber that was attached to the X-ray tube head and a set-top box displaying the DAP value. The measurement was performed 3 times and averaged to minimize error. The values were calibrated with temperature and pressure coefficients before being averaged.

#### Reference point

The reference point is the point where the central X-ray from all projection angles intersects, and it is shown in terms of X, Y, and Z coordinates. The X-axis crosses from left to right, the Y-axis from posterior to anterior, and the Z-axis from inferior to superior (Fig. 2a). The reference point was determined by the program to be (0, −3, 47), corresponding to the centre of the dental arch. The centre of the whole body was (0, 0, 0).

#### X-ray tube voltage

The X-ray tube voltage was 66 kVp, the same as the TLD measurement condition.

The input values for factors related to panoramic machine geometry followed the manufacturer’s specifications and previous studies in the literature that used the same machine (Fig. 2a)^9^.

#### Rotation angle

The rotation angle is the angle at which the X-ray source and the film rotate. The input value was 240°, according to the manufacturer’s specifications.

#### Vertical angle

The vertical angle is defined as the vertical angle formed by the central ray, and a value of −8° was entered.

#### FRD

A value of 35 cm was input for the distance from the X-ray source to the reference point.

#### Beam width and height

Beam width and height at the reference point were calculated based on the collimator size, FRD, and focus-to-collimator distance (FCD) (Fig. 2b)^9^. Values of 0.20 cm and 10.00 cm were input for the beam width and height, respectively. The equations for the calculation were as follows:

$$Beam\,Height=\frac{Collimator\,Height\,X\,FRD}{FCD},$$

$$Beam\,Width=\frac{Collimator\,Width\,X\,FRD}{FCD},$$

where collimator height = 3.78 cm, collimator width = 0.09 cm, FCD = 13.0 cm, and FRD = 35.0 cm. These values were obtained from the manufacturer’s specifications and manual measurements made using digital callipers.

#### Filtration

A value of 2.8 mmAl was input for filtration according to the manufacturer’s specifications.

### Impact of individual factors on the effective dose

To assess the impact of each factor on the effective dose, the effective doses were repeatedly calculated by applying different values for each factor. When various values were input for a given factor, the other factors were fixed to the general conditions described above.

The minimum and maximum input values were determined for the rotation angle, vertical angle, FRD, X-ray beam width and height, filtration, and X-ray tube voltage based on the manufacturer’s specifications. Then, within this range, 6 input values were determined, at even intervals (Table 3). For factors that did not allow varied input values, including patient age, the reference point, and input dose, the standard values were used.

**Table 3:** The six varied input values for individual factors in the Monte Carlo simulations.

Factor	Varied input values					
Rotation angle (°)	180	192	204	216	228	240
Vertical angle (°)	−10	−9	−8	−7	−6	−5
FRD (cm)	25	27	29	31	33	35
Beam width (cm)	0.20	1.06	1.92	2.78	3.64	4.50
Beam height (cm)	4.80	5.84	6.88	7.92	8.96	10.00
Filtration (mmAl)	2.51	2.57	2.63	2.69	2.75	2.81
X-ray tube voltage (kVp)	57	60	63	66	69	72

FRD, focus-to-reference distance.

#### Rotation angle

The obtained panoramic image length measured with a digital calliper in the image viewer was 240.0 mm with 240° of rotation (Fig. 2c). The minimum projection angle was determined as 180°, from which a 180.0-mm length of image for a 5-year-old patient can be obtained (Fig. 2c). Rotation angles of 180°, 192°, 204°, 216°, 228°, and 240° were used.

#### Vertical angle

The vertical angle of the X-ray beam in panoramic radiography is known to range between −5° and −10°, so values of −5°, −6°, −7°, −8°, −9°, and −10° were input for this factor.

#### FRD

The patient’s position should not be closer to the source than the midpoint of the film-to-source distance (FSD) to obtain the image, considering the mechanical geometry of panoramic machines. Thus, the minimum FRD was determined as 25 cm, which was the half of the total FSD measured manually. The FRD was input with values of 25, 27, 29, 31, 33, and 35 cm.

#### Beam width and height

According to a report published by the Korean Ministry of Food and Drug Safety in 2014, collimator width was 0.2–4.5 mm and collimator height was 4.8–10.1 mm^16^. For the beam size calculation equation described above, the input values for beam width were 0.2, 1.06, 1.92, 2.78, 3.64, and 4.5 cm and those for beam height were 4.80, 5.84, 6.88, 7.92, 8.96, and 10.0 cm^9^.

#### Filtration

According to the 2014 report, in Korea, the mean filtration value was 2.66 ± 0.15 mmAl^16^. Thus, input values of 2.51, 2.57, 2.63, 2.69, 2.75, and 2.81 mmAl were used.

#### Tube voltage

The input values ranged from 57 to 72 kVp for this factor. The lowest available tube voltage for the machine was 57 kVp. To determine the maximum tube voltage, it was considered that male adults would be exposed to 73 kVp. Therefore, values of 57, 60, 63, 66, 69, and 72 kVp were input for this factor.

### Data analysis

Statistical analysis was performed using IBM SPSS^®^ Statistics version 23 (IBM Corp., Armonk, NY, USA). Simple linear regression analysis was applied to evaluate the influence of individual factors on the effective dose. The coefficient of determination (R^2^) was obtained to verify the fit of this analysis. Regression coefficients were obtained to compare the impact of each factor on the effective dose.

## Results

**Figure 3:**
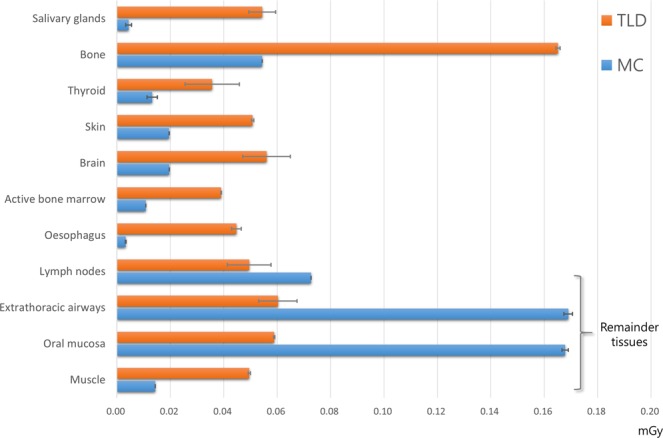
Organ-absorbed dose measured with thermoluminescent dosimetry (TLD) and simulated with the Monte Carlo method.

The effective dose measured using TLD was 3.850 µSv, while the MC-simulated effective dose was 3.474 µSv in the general conditions. The TLD recorded value of individual anatomic site was obtained for the organ-absorbed dose measurement (Supplementary Table 1s). The organ-absorbed dose measured with TLD and PCXMC is illustrated in Fig. 3. Both the TLD measurements and the MC simulation showed the highest organ dose in the bone, except for the remainder tissues. Among the remainder tissues, both TLD and PCXMC showed the highest value for the extrathoracic airways.

**Figure 4:**
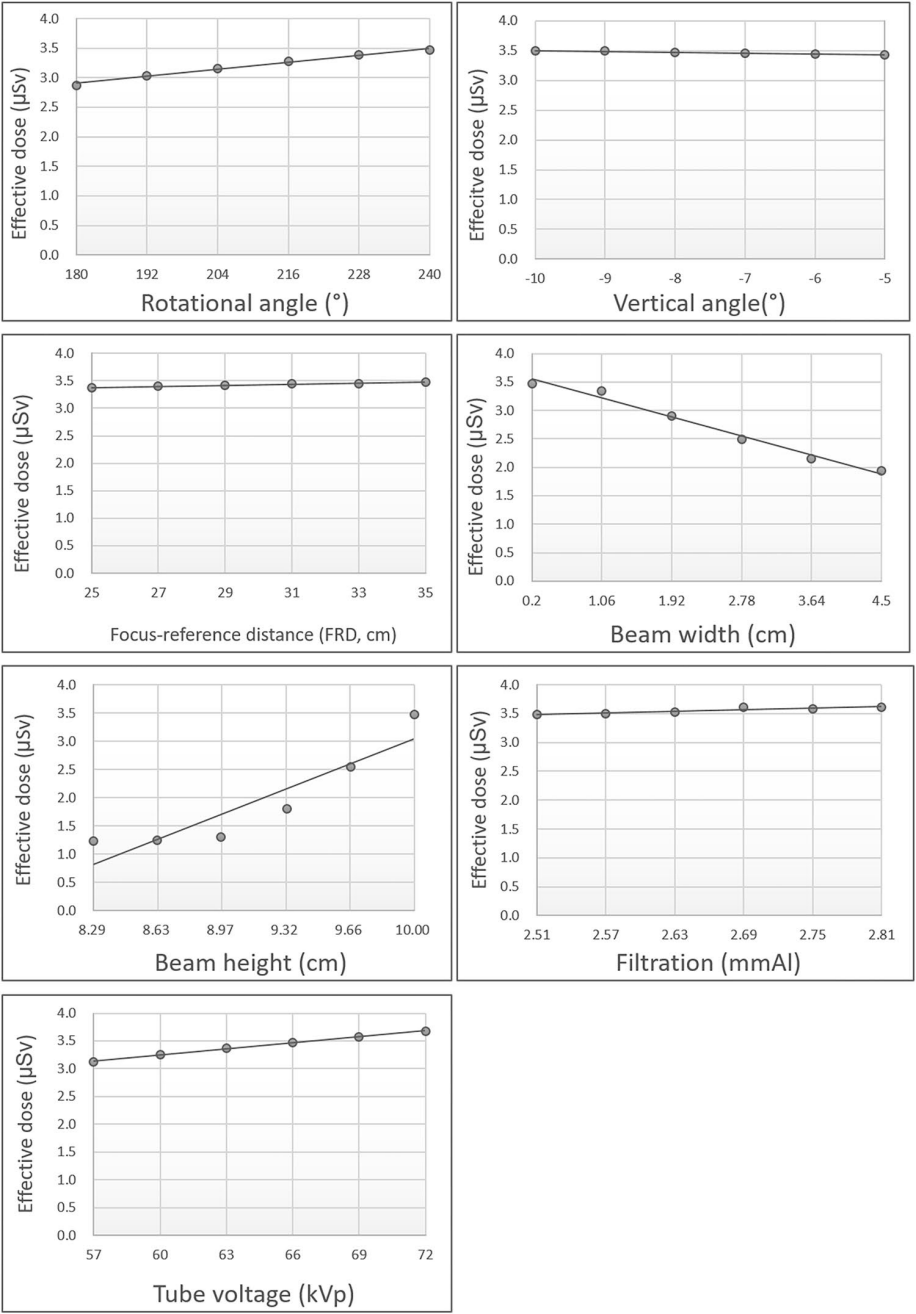
The influence of individual factors on the effective dose using the Monte Carlo method. Factors with steep slopes are considered to have a significant effect on the effective dose.

All coefficients of determination (R^2^) were close to 1 and the regression model was well suited for describing the relationship between the factors and the effective dose. The tube voltage was best fitted model (R^2^ = 0.9987) and the beam height was the relatively moderate fitted model (R^2^ = 0.8840) for the effective dose. The rotation angle, FRD, beam height, filtration, and tube voltage showed significant positive correlations with the effective dose, while vertical angle and beam width showed significant negative correlations (P < 0.05) (Fig. 4). According to the regression coefficient values, beam height had the greatest impact on the effective dose, while rotation angle and FRD had the smallest impact (Table 4).

**Table 4:** The impact of dose-determining factors in the Monte Carlo method for effective dose calculation analysed with simple linear regression.

factor	Coefficient of determination (R^2^)	Regression coefficient	*p-*value
Rotation angle	0.9903	0.0001	0.000
Vertical angle	0.9557	−0.0002	0.001
Beam width	0.9827	−0.0039	0.000
Beam height	0.8440	0.0130	0.010
FRD	0.9583	0.0001	0.001
Filtration	0.8907	0.0046	0.005
X-ray tube voltage	0.9987	0.0004	0.000

FRD, focus-to-reference distance.

## Discussion

MC simulation is a relatively uncommon method of obtaining the effective dose in dentistry, although it is widely used in the medical field due to its simplicity and high accuracy. Recently, the MC method has been reported to show clinically acceptable results in dental cone-beam computed tomography (CBCT) compared to the traditional method using TLD^8^. Additionally, in panoramic radiography units, for which the principles of machine operation are much more complex than those of CBCT, the MC method showed comparable results to TLD for effective dose acquisition when appropriate input values were used in the simulation^8^. The present study also verified the reliability of MC simulation for effective dose acquisition in paediatric panoramic radiography. When compared with TLD (3.850 µSv), the effective dose estimated using the MC simulation (3.474 µSv) showed less than a 10% difference. The recent study, compared MC simulation with TLD method, also mentioned that MC simulation is reliable as it showed agreement within ±10.1% with the TLD result^8^. Based on its high precision and simplicity in use, it was possible to estimate the effective dose in 36 different conditions through MC simulations. Repeated measurements would have been challenging if TLD with an anthropomorphic phantom was used. In this study, approximately 4 days were spent when using TLD method for measuring radiation dose of panoramic radiography. On the other hand, it took about 30 minutes to an hour for MC simulation to present the organ absorbed dose and the effective dose as a result. Thus, it is expected that MC simulations will help clinicians easily simulate their own ideas for dose reduction methods with their own panoramic radiography machines.

Even though MC simulation was found to be reliable in previous studies for obtaining the effective dose, controversies remain about individual organ-absorbed dose calculations^8,9,17^. This study also showed discrepancies in the individual organ dose between the 2 different methods, similar to the previous research reported by Lee *et al*.^9^. However, a more recent study on dental CBCT reported that the discrepancy in the organ dose between the TLD and MC methods was only ±10.1%. They used the same anthropomorphic phantom for both methods. To adopt the same phantom in the MC method, they used computed tomography scan data of the phantom used in the TLD method. Zang *et al*. stated that the virtual phantom used in an MC simulation is an important factor for ensuring precise organ dose calculations^18^. However, any human phantom is different from individual human beings. In addition, an international consensus is needed to develop phantoms that can be used widely. Also, the error of the TLD measurement method is expected to contribute to the difference of these measured values. As mentioned in the previous literature, sampling error of TLD method may result in a great times of overestimation, especially in absorption dose of body-wide distributed organs^19^. Thus, it is suggested that relative tendencies should be evaluated, instead of comparing absolute values. Both TLD and MC simulations showed a higher dose for the brain than for the salivary gland or thyroid gland, which contradicts the results of previous studies for adult patients^9^.

Overall, evaluating the effective dose is important for paediatric patients. In general, 6 years is the initial age when children show transitional dentition from deciduous to permanent dentition. Between the ages of 5 and 8 years, dental arch width rapidly grows, especially in the molar region, as permanent teeth start erupting^20^. The American Academy of Paediatric Dentistry recommends panoramic radiography for children with transitional dentition in its guideline^21^.

Therefore, many studies have investigated the effective dose of dental panoramic radiography, and it has been reported to range widely, from 3.85 to 38.00 µSv^5,11,22,23^. According to Hayakawa and colleagues, effective doses were diverse due to the different geometries and exposure conditions of the machines^5^. To confirm which factors contribute most strongly to the resulting effective dose, dose measurement experiments should be performed repeatedly with different machine operational geometries and exposure conditions.

In this study, the DAP, reference point, and X-ray tube voltage were thought to be factors influencing X-ray exposure conditions in paediatric panoramic radiography. Of these factors, X-ray tube voltage is the only adjustable factor, and different values were simulated to calculate the effective dose. The factors related to machine operational geometry, rotation angle, vertical angle, FRD, beam width, height, and filtration cannot be modified by clinicians. However, it is important to understand the impact of those factors on the effective dose, so that clinicians may choose a machine appropriately or use a low-dose examination mode.

Reducing the rotation angle helped to diminish the effective dose. As the rotation angle of the beam decreased, some organs were less included in the radiation-exposed area. Since the dental arch in children is smaller than in adults, a small rotation angle is expected to cover enough area to obtain a usable image. According to a clinical image quality evaluation chart, the coverage area of panoramic radiography on the left and right sides is 0.5 mm lateral to the temporomandibular joint^24^. Paediatric panoramic radiography generally includes more lateral regions than are imaged in adults. Further study is needed for reducing the unnecessarily exposed area by decreasing the rotation angle.

The vertical angle of the central X-ray beam also showed a negative relationship with the effective dose. Since the vertical angle is a negative value, as the source is located below the object and the central ray goes upward, it can be inferred that the absolute value of the vertical angle showed a positive relationship with the effective dose. As the central X-ray was projected with a more vertical angle, the effective dose became greater. This was probably due to the fact that the central ray became closer to the organs with a greater impact on the effective dose.

Beam width showed a negative relationship with the effective dose. Lee *et al*. indicated that photon density became dispersed as the beam size increased with the total number of photons remaining constant^9^. Although beam width had a strong influence on the effective dose, its impact was not great as that of beam height. X-ray beam height had the greatest influence on the effective dose.

Applying a short-height beam by decreasing the collimator height to reduce the effective dose has been reported previously. Davis *et al*. reported that the effective dose decreased by 32% when the collimator height was reduced by 30 mm^4^. It is interesting that beam height shows opposite effects on the effective dose between adult and paediatric patients. Additionally, beam height showed a relatively uneven effect on the effective dose, and it is suspected that complex physical and biological factors shape the influence of beam height on the effective dose. According to a previous study of MC simulation for adult panoramic radiography, the effective dose slightly decreased when the beam height was increased^9^. Meanwhile, another specific biological factor should be considered for paediatric panoramic radiography. In 2013, Ludlow *et al*. reported that the thyroid gland is more closely located to the mandible in children, leading to a significant increase in the overall effective dose^6^. Thus, the different effects of beam height between adult and paediatric patients were probably due to differences in the location and size of head and neck organs. In fact, when the beam height was increased from 9 cm to 10 cm, the dose of paediatric patients increased by about 30% in our study, while adults showed a decrease of approximately 2% in the previous study^9^. Therefore, a panoramic machine with a short collimator height should be used, especially for paediatric patients. Of note, some panoramic machines are equipped with a paediatric collimator, which should be considered as an important factor when selecting a machine for paediatric dentistry.

The FRD showed a positive correlation with the effective dose. X-ray photons undergo scattering as they pass through the air. When the FRD increases, photon scattering also increases, inevitably increasing the patient dose. Thus, when using paediatric mode in panoramic radiography, it would be helpful to reduce the FRD in order to diminish the effective dose. Nonetheless, FRD (similarly to the rotation angle, as discussed above), did not show a substantial influence on the effective dose compared to the other factors.

X-ray tube voltage and filtration showed positive correlations with the effective dose. Marin *et al*. reported that the effective dose increased when the tube voltage was increased^25^. They measured the effective dose of multidetector abdominal computed tomography with a metal-oxide semiconductor transistor and a human body phantom in 2 different tube voltage conditions. However, theoretically, when the tube voltage is increased, the effective dose should decrease, since there are fewer low-energy photons in the X-ray. The same reasoning can be applied to filtration. When filtration increases, there are fewer low-energy photons in the X-ray beam. Low-energy photons are known to be harmful to patients because they are absorbed by tissues. Thus, further study is required to confirm the actual impact of tube voltage and filtration on the effective dose in the human head and neck region.

This study attempted to compare the accuracy of MC simulation to that of the conventional TLD method. It has been suggested that MC simulation presented comparable result as TLD method in effective dose calculation. In spite, since this was the first study to adopt MC method on paediatric panoramic radiography, further evaluation with various models of panoramic radiography would be needed. Additionally, various dose-influencing factors were modified and simulated to determine which had a major impact on reducing the effective dose. However, this study did not consider the overall image quality. Further study is needed to achieve adequate image quality while reducing the effective dose.

In conclusion, MC simulation is comparable to the TLD method for obtaining effective dose estimates in paediatric panoramic radiography, and it is clinically applicable. Obtaining panoramic radiography with a short beam height can effectively reduce the dose in paediatric patients.

## Supplementary information

Titlepage, Supplementary Table 1s
