# Supplementary material for: Efficacy of the Monte Carlo method and dose reduction strategies in paediatric panoramic radiography
Source: Sci Rep. 2019 Jul 4;9:9691. doi: 10.1038/s41598-019-46157-0 (PMC6609601; doi:10.1038/s41598-019-46157-0)
Supplement: Supplementary file 1 — Titlepage, Supplementary Table 1s [file 41598_2019_46157_MOESM1_ESM.docx]

**Titlepage**

**Efficacy of Monte Carlo method and dose reduction strategies in paediatric panoramic radiography**

Chena Lee, Bora Park, Sam-Sun Lee^*^, Jo-Eun Kim, Sang-Sun Han, Kyung-Hoe Huh, Won-Jin Yi, Min-Suk Heo, Soon-Chul Choi

Chena Lee. DDS, PhD. Assistant professor of clinical research, Department of Oral and Maxillofacial Radiology, Yonsei University College of Dentistry, Seoul, Republic of Korea

Bora Park, DDS. Graduate student, Department of Oral and Maxillofacial Radiology and Dental Research Institute, School of Dentistry, Seoul National University, Seoul, Republic of Korea

Sam-Sun Lee, DDS, PhD. Professor. Department of Oral and Maxillofacial Radiology and Dental Research Institute, School of Dentistry, Seoul National University, Seoul, Republic of Korea

Jo-Eun Kim, DDS, PhD. Clinical professor. Department of Oral and Maxillofacial Radiology and Dental Research Institute, School of Dentistry, Seoul National University, Seoul, Republic of Korea

Sang-Sun Han. DDS, PhD. Associate professor, Department of Oral and Maxillofacial Radiology, Yonsei University College of Dentistry, Seoul, Republic of Korea

Kyung-Hoe Huh, DDS, PhD. Professor, Department of Oral and Maxillofacial Radiology and Dental Research Institute, School of Dentistry, Seoul National University, Seoul, Republic of Korea

Won-Jin Yi, Professor, DDS, PhD. Department of Oral and Maxillofacial Radiology and Dental Research Institute, School of Dentistry, Seoul National University, Seoul, Republic of Korea

Min-Suk Heo, Professor, DDS, PhD. Department of Oral and Maxillofacial Radiology and Dental Research Institute, School of Dentistry, Seoul National University, Seoul, Republic of Korea

Soon-Chul Choi, Professor, DDS, PhD. Department of Oral and Maxillofacial Radiology and Dental Research Institute, School of Dentistry, Seoul National University, Seoul, Republic of Korea

Correspondence to: Prof. Sam-Sun Lee

Department of Oral and Maxillofacial Radiology, School of Dentistry, Seoul National University, 101 Daehak-ro, Jongno-gu, Seoul 03080, Korea

Tel: 82-2-2072-3978, Fax: 82-2-744-3919, E-mail: raylee@snu.ac.kr

Word Count: 3,678 words

Supplementary Table 1s. Average and standard deviation of absorbed dose of each anatomic site TLD placed

| Anatomic site | TLD chip ID | | | Average | Standard deviation |
| --- | --- | --- | --- | --- | --- |
|  | Dose (µGy) | | |  |  |
| Anterior calvarium | H3 | H7 | H8 |  |  |
|  | 94.48 | 128.10 | 115.30 | 112.63 | 16.97 |
| Left calvarium | G4 | G11 | G12 |  |  |
|  | 54.26 | 58.75 | 87.68 | 66.90 | 18.14 |
| Right calvarium | F6 | G3 | F12 |  |  |
|  | 121.60 | 74.11 | 121.00 | 105.57 | 27.25 |
| Midbrain | E2 | E3 | D2 |  |  |
|  | 89.15 | 59.24 | 136.50 | 94.96 | 38.96 |
| Midbrain | E11 | F5 | F2 |  |  |
|  | 111.70 | 81.77 | 87.21 | 93.56 | 15.94 |
| Pituitary / Left nasopharynx | K3 | K6 | K12 |  |  |
|  | 300.60 | 301.90 | 344.50 | 315.67 | 24.98 |
| Right maxillary sinus | B3 | B4 | B9 |  |  |
|  | 92.14 | 125.80 | 122.20 | 113.38 | 18.48 |
| Right ramus / parotid gland / submandibular gland | AS1 | AS4 | AS7 |  |  |
|  | 246.90 | 209.90 | 202.00 | 219.60 | 23.97 |
| Left ramus / parotid gland / submandibular gland | R1 | P6 | Q1 |  |  |
|  | 168.00 | 178.10 | 176.20 | 174.10 | 5.37 |
| Esophagus | A6 | L2 | L3 |  |  |
|  | 192.60 | 125.40 | 84.53 | 134.18 | 54.57 |
| Center sublingual gland | O7 | O4 | O8 |  |  |
|  | 80.17 | 96.15 | 112.00 | 96.11 | 15.92 |
| Right lens of eye | B3 | B4 | B9 |  |  |
|  | 92.14 | 125.80 | 122.20 | 113.38 | 18.48 |
| Left lens of eye | C6 | C11 | C7 |  |  |
|  | 137.80 | 58.94 | 125.00 | 107.25 | 42.32 |
| Left back of neck | AS10 | J3 | J8 |  |  |
|  | 253.50 | 239.90 | 217.00 | 236.80 | 18.45 |
| Left Thyroid | N1 | H10 | M7 |  |  |
|  | 80.52 | 113.50 | 84.86 | 92.96 | 17.92 |
| Right Thyroid | M1 | M6 | H9 |  |  |
|  | 120.60 | 87.68 | 196.30 | 134.86 | 55.70 |
| Center C spine | O1 | O3 | N2 |  |  |
|  | 84.20 | 51.78 | 42.71 | 59.56 | 21.81 |
